# Comparative Immunomodulatory Evaluation of the Receptor Binding Domain of the SARS-CoV-2 Spike Protein; a Potential Vaccine Candidate Which Imparts Potent Humoral and Th1 Type Immune Response in a Mouse Model

**DOI:** 10.3389/fimmu.2021.641447

**Published:** 2021-05-24

**Authors:** Tripti Shrivastava, Balwant Singh, Zaigham Abbas Rizvi, Rohit Verma, Sandeep Goswami, Preeti Vishwakarma, Kamini Jakhar, Sudipta Sonar, Shailendra Mani, Sankar Bhattacharyya, Amit Awasthi, Milan Surjit

**Affiliations:** Infection and Immunology, Translational Health Science & Technology Institute, National Capital Region (NCR) Biotech Science Cluster, Faridabad, India

**Keywords:** SARS-CoV2, RBD, spike protein, vaccine, immunogenicity, immunomodulation

## Abstract

The newly emerged novel coronavirus, SARS-CoV-2, the causative agent of COVID-19 has proven to be a threat to the human race globally, thus, vaccine development against SARS-CoV-2 is an unmet need driving mass vaccination efforts. The receptor binding domain of the spike protein of this coronavirus has multiple neutralizing epitopes and is associated with viral entry. Here we have designed and characterized the SARS-CoV-2 spike protein fragment 330-526 as receptor binding domain 330-526 (RBD_330-526_) with two native glycosylation sites (N331 and N343); as a potential subunit vaccine candidate. We initially characterized RBD_330-526 _biochemically and_ _investigated its thermal stability, humoral and T cell immune response of various RBD protein formulations (with or without adjuvant) to evaluate the inherent immunogenicity and immunomodulatory effect. Our result showed that the purified RBD immunogen is stable up to 72 h, without any apparent loss in affinity or specificity of interaction with the ACE2 receptor. Upon immunization in mice, RBD generates a high titer humoral response, elevated IFN-γ producing CD4+ cells, cytotoxic T cells, and robust neutralizing antibodies against live SARS-CoV-2 virus. Our results collectively support the potential of RBD_330-526_ as a promising vaccine candidate against SARS-CoV-2.

## Introduction

Emerging and reemerging pathogens are always a threat and a challenge for society and public health globally. The outbreak of a novel coronavirus, severe acute respiratory syndrome coronavirus 2 (SARS-CoV-2) in Wuhan, China, in December 2019 with an unidentified form of viral pneumonia, has stirred a global public health crisis affecting more than 235 countries and territories worldwide. SARS-CoV-2 was identified as the seventh member within the family “*coronaviridae*”, which has crossed the species barrier to infect humans (1). SARS-CoV-2 belongs to the beta genus of the *coronaviridae* family. The other two previously known members of the beta coronavirus genus are severe acute respiratory syndrome coronavirus (SARS-CoV) and Middle East respiratory syndrome coronavirus (MERS-CoV) which were responsible for the outbreaks in 2002-2003 and 2012 respectively and have been linked to fatal illness (2).

The SARS-CoV-2 viral genome shares 79.5% nucleic acid sequence identity with SARS-CoV (3) and 96.2% with that of a bat coronavirus (RaTG13) (3, 4). MERS, SARS-CoV, and SARS-CoV-2 are believed to have originated from bats and the zoonotic intermediate for transmission to humans include dromedary camels in MERS and palm civets and raccoon dogs for SARS-CoV, while the intermediate host for SARS-CoV-2 still remains unknown (5). The high prevalence of coronaviruses and SARS-related coronaviruses, wide distribution, genetic diversity, and increasing human–animal interface activities, and the recurrent spillovers of coronavirus may suggest that in the future novel coronaviruses are likely to emerge periodically affecting humans and probably causing future epidemics (6, 7). Hence, urgent development of safe and effective vaccines is needed to prevent and reduce the spread of this outbreak, public health burden, and hopefully prevent such incidents in the future. As per WHO, more than 180 vaccine candidates are currently in various stages of research and development across the world, utilizing different platforms such as inactive virus, mRNA, protein subunits, replicating viral vector, and virus-like particles (VLP).

As of December 29, 2020, approximately 1.7 million people (WHO) have lost their battle to this pandemic around the world (8). SARS-CoV-2 virus infection leads to characteristic respiratory distress symptoms among COVID-19 patients (9). The virus enters human cells through the interaction of host receptor angiotensin-converting enzyme 2 (ACE2) and the receptor binding domain (RBD) of the spike protein, which further leads to the fusion of two membranes. The fusion process involves cleavage at the interface of S1–S2 and S2′ sites with the S1 subunit of the spike protein binding to the host cell receptor and S2 playing a role in viral and cellular membrane fusion. The spike protein consists of two conformations; symmetric, with all RBDs in the down position, and asymmetric homotrimeric conformation in which one or two protomers among the trimer have an RBD in the up or erect position. An asymmetric conformation is designated as an inactive or non-ACE2 binding position, a symmetric conformation is considered to be an ACE2 binding conformation. However, the overall structure of the SARS-CoV-2 spike protein is similar to the SARS-CoV spike protein trimer (10–12). Unlike the influenza HA and HIV gp160 (gp120-gp41) trimers, SARS-CoV-2 exhibits a dynamic conformation between ACE2 binding and nonbinding states with infrequent exposure of its receptor binding domain (RBD) (13). The viral entry of SARS-CoV-2, similar to SARS-CoV, is dependent on a binding interaction between the RBD of the viral spike protein and ACE2 on the host cell surface. However, only 47.8% of sequence similarity is reported between the RBD of the two viruses (14). Despite this, it has been reported that SARS-CoV-2 RBD binds to ACE2 with much greater affinity (factor of 5-10-fold) (15), as well as the fact that S1–S2 furin cleaving site “RRAR” of SARS-CoV-2 represents a similar match to cellular serine protease TMPRSS2 (16). Recently published reports also highlight the possible involvement of other coreceptors which may be crucial for cellular entry and infectivity of SARS-CoV-2 and Neuropilin-1 (NRP-1) (17). These combining factors are believed to contribute to the efficiency of virus transmission, making COVID-19 more contagious than other respiratory diseases like SARS-CoV and influenza.

Previous studies have indicated that the RBD of the spike protein of the coronavirus contains multiple conformational neutralizing epitopes and is a vulnerable target for nAbs (18–20). MERS and SARS RBD tend to have more neutralizing potential than other epitopes of the protein. However, SARS-CoV ACE2 binding site-directed antibodies (m396, CR3019) fail to bind SARS-CoV-2; suggesting the changes in structure and its composition contributes towards the uniqueness and importance of the SARS-CoV-2 RBD (1). The RBD of the SARS-CoV-2 spike protein is critically defined as the most likely target for the development of virus attachment inhibitors, neutralizing antibodies, and vaccines. Hence, in response to the urgent prophylactic measures needed against SARS-CoV-2, it is necessary to understand the potential of mounted immune response in providing protection. Therefore, in an effort directed toward the development of a safer subunit vaccine, here we expressed the non-truncated wild-type sequences of SARS-CoV-2 as an RBD through the mammalian expression system. We have characterized its biochemical characteristics and investigated the immunomodulatory response in the presence and absence of adjuvants. We attempt to propose an ideal vaccine candidate with safety and high neutralization potential as an aid to support the ongoing combat against SARS-CoV-2.

## Material and Methods

### Materials

Expi293 cells (Expi293F™ cells originated from parental FreeStyle™ 293-F cells, catalog number: A14527) was obtained from Thermo Fisher Scientific. HEK293T, Huh-7, and Vero-E6 cells were obtained from the American Type Culture Collection (ATCC). Anti-SARS polyclonal antibody (Anti-SARS-pAb) and SARS/SARS-CoV-2 coronavirus spike protein (subunit 1) polyclonal antibody (catalog number; PA5-81795, Invitrogen) were obtained from Thermo Fisher Scientific. CR3022 monoclonal anti-RBD antibody (clone CR3022, produced in *Nicotiana benthamiana*, contributor: Novici Biotech LLC) was obtained from BEI Resources (NR-52392). Adjuvants AddVax (AddaVaxTM squalene-oil-in-water, 10 ml; catalog number: vac-adx-10) was bought from InVivoGen (USA) and Imject (Imject™ Alum Adjuvant; catalog number: 77161) was obtained from Sigma (USA). SARS-CoV-2 virus was deposited by the Centers for Disease Control and Prevention and obtained through BEI Resources, NIAID, NIH; SARS-related coronavirus 2, isolate USA-WA1/2020 (NR-52281). Peroxidase AffiniPure goat anti-mouse IgG (H+L) (catalog number: 109-035-088), peroxidase AffiniPure goat anti-rabbit IgG (H+L) (catalog number: 111-035-144), and peroxidase AffiniPure goat anti-human IgG (H+L) (catalog number: 109-035-088) were obtained from Jackson Immuno Research (USA). ACE2 monoclonal antibody (catalog number: MA531395) was obtained from Thermo Fisher Scientific. Antibodies for immunofluorescence assays; anti-mouse Alexa Fluor 488 (Catalog Number: A32723), goat anti flag AlexaFlour 647 (Catalog number: MA1-142-A647), and ProLong Gold anti-fade reagent with DAPI (Catalog number: P36941) were obtained from Thermo Fisher Scientific. Antibodies for FACS studies; PerCP anti-mouse CD4 antibody (clone: RM4-5, catalog number: 100538), FITC anti-mouse CD8 antibody (clone: 5H10-1, catalog number: 100804), PE anti-mouse IL-2 antibody (clone: JES6-5HM, catalog number: 503808), PE/Cyanine7 anti-mouse IL-17A antibody (clone: TC11-18H10.1, catalog number: 506922), and Alexa Fluor^®^ 647 anti-mouse IFN-γ antibody (clone: XMG1.2, catalog number: 505814) were obtained from Biolegend, USA.

### Sequence Analysis of SARS-CoV-2 and Alignment

The SARS-CoV-2, severe acute respiratory syndrome coronavirus 2 isolate 2019-nCoV WHU01, (GenBank: MN988668.1, release date February 11, 2020, spike protein; GenBank: QHO62107.1) sequence was analyzed for the identification of RBD coding sequences. Blastp (protein-protein blast) was performed with SARS-CoV-2 to the spike protein amino acid sequence against the PDB database. The closest matched 6ZGF with 89.34% was the spike protein of RaTG13 bat coronavirus in a closed conformation (21), we further analyzed the hit to ascertain whether the structure corresponded to receptor binding domains 2GHV (73.60%) (18), 2DD8 (74.11%) (19), and 3BGF (73.71%) in sequence identity. All the respective sequences belong to severe acute respiratory syndrome coronavirus. Multiple sequence alignment was performed using Clustal Omega and represented through Esprit (22). The N-linked glycosylation sites were predicted through the NetNGlyc 1.0 server (23). The structural superimposition was performed through Pymol. Receptor binding motif (RBM) and cysteine bonds were identified through the modeled structure of SARS-CoV-2 (swiss modeler) which was further validated in light of available structures.

### Cloning, Transient Expression, and Purification of SARS-CoV-2 RBD

The coding sequences for the receptor binding domain (RBD) of the spike protein from the SARS-CoV-2 isolate was codon-optimized from GeneArt (Thermofisher Inc.) for expression in the mammalian expression system. The RBD expressing 330 to 526 amino acids of the SARS-CoV-2 spike protein (_330_PNITNLCPF to HAPATVCG_526_ amino acid) along with CD5 secretory sequences at the N-terminal and a GSGG linker at the C-terminal followed by six histidine residues was cloned into mammalian expression vector pCDNA3.1(+) (Invitrogen) in the *BamH*I and *Xho*I restriction sites.

The recombinant protein was expressed and purified from Expi293 cells (ThermoFisher) by utilizing the manufacturer’s protocol. Briefly, Expi293 cells were transfected with purified DNA using an Expi Fectamine 293 Transfection Kit (ThermoFisher). Supernatants with secreted protein were harvested 6 days post-transfection by centrifugation of the culture. The harvested supernatant with RBD was loaded onto Ni-NTA agarose (Qiagen), equilibrated with 50 mM of Tris (pH 7.4) and 100nM of NaCl at 4°C utilizing gravitational flow, the column was further washed and protein was eluted with elution buffer containing 500 mM of imidazole. The eluted factions from the Ni-NTA column were checked on SDS PAGE, Ni-NTA-purified fractions were then pooled and concentrated using Amicon centricons (10 kDa cutoff) and further purified through size exclusion chromatography using a Superdex 200 Increase 10/300 GL column (GE Healthcare), equilibrated in PBS. The RBD was eluted as a monomer and the purified RBD was snap-frozen in liquid nitrogen and stored at −80°C until further use.

### Gel Filtration Chromatography

Gel filtration chromatography was performed using a Superdex S-200 10/300 GL analytical column calibrated with a Gel Filtration Calibration Kit (HMW) to evaluate the oligomeric status of the purified proteins and to separate and purify the mixed oligomeric population and trace contaminants. The pre-equilibrated column in PBS was used for the analysis of the RBD-ACE2 complex, a peak integration module was used to identify the elution peak position of the purified proteins.

### Western Blot Analysis

The protein samples for analysis were run on 12% of SDS-PAGE for RBD and on 8% for the spike pre-fusion trimer (S2P). The gel was run for 2 h at 120 Volts for the proper resolution of protein. Resolved proteins in gel were then transferred to a PVDF membrane. The membrane was blocked with 5% skimmed milk, incubated with primary Ab (1:5000; pooled sera from RBD immunized groups, SARS/SARS-CoV-2 coronavirus spike protein (subunit 1) polyclonal antibody; 1:1000, anti His antibody; 1:1000), incubated overnight. The membrane was incubated with HRP conjugated anti-mouse (1:2000 for anti RBD sera and anti His blots) and anti-rabbit secondary (1:2000 for SARS polyclonal sera) antibodies and developed using Femtolucent Plus HRP (G Biosciences).

### Nano DSC

Thermo-stability of RBD was analyzed with a Nano-DSC (TA instruments) as described previously (24, 25). Briefly, the protein sample in PBS was concentrated at 2.2 mg/ml for measurement. Thermal melting was performed at a scanning rate of 1°C/min under 3.0 atmospheres of pressure. A similar scan was performed with the buffer (PBS) for base line correction. Data collected were analyzed with NanoAnalyze software, 3.11.0 (TA instruments), with buffer correction, subtraction, and normalization.

### Antigen ELISA

The ELISA was performed to characterize the binding of monoclonal antibody CR3022, ACE2-Fc, and SARS/SARS CoV-2 polyclonal sera to RBD as described previously (26), Briefly, Maxisorp plates (Nunc) were coated with 100 µl of protein (2 µg/ml concentration) in 1x carbonate/bicarbonate buffer, pH 9.6 overnight at 4°C. The following day, the plates were blocked using 250 µl of PBS containing 5% skimmed milk (blocking buffer). The antibody/protein was diluted serially 1:3 times in the dilution buffer with a starting concentration of 10 mg/ml, incubated, and processed further as described (26). HRP-conjugated anti human (1:10,000 dilution, Jackson Immuno Research, USA) and anti rabbit (1:5,000 dilution, Jackson Immuno Research, USA) antibodies were used for CR3022, ACE2-Fc, and SARS/SARS CoV polyclonal sera as the secondary antibodies.

### BLI

Binding kinetics assays were done using an Octet Red96 instrument (ForteBio, Inc., USA). The binding of monoclonal antibody CR3022 to RBD was performed using anti-human Fc sensors (AHC). CR3022 at a concentration of 10 μg/ml were captured on AHC sensors for 150 s. RBD as an analyte was 3-fold serially diluted in kinetic buffer (PBS with 0.02% Tween 20 and 0.1% BSA) and 200 ul of diluted analyte was dispensed into 96-well microtiter plates per well. Antibodies immobilized sensors were immersed in diluted analytes for 90 s to record association. The dissociation was recorded by transferring the sensors to wells containing buffer for 180 s.

For biotinylated hACE2-RBD interactions, the biotinylated hACE2 were immobilized on a streptavidin-coated biosensor surface (SA). A set of reference sensors (SA) were blocked using biotinylated BSA (50 µg/ml) to reduce non-specific background binding. RBD as an analyte at 210 nM concentration was dispensed into 96-well microtiter plates per well. The association was performed for 400 s and dissociation for 600 s.

Data were collected and analyzed using ForteBio Data Analysis software, 9.0 (ForteBio Inc). A 1:1 global curve-fitting model was used for the analysis of antibodies with one to one binding stoichiometry. Whole experimental procedures were performed at RT. The fittings were considered to be satisfactory if χ2 < 0.5. Standard deviation was calculated from three independently performed experiments.

### Ethics Statement

Animal studies were performed as per Institutional Animal Ethics Committee (IAEC) approval. The mice immunization studies were performed under the approval number IAEC/THSTI/93; project entitled “Immunogenicity Assessment of SARS-CoV-2 spike protein-based immunogen” following protocols for the care and use of laboratory animals. The animal immunization studies were performed at the Small Animal Facility (SAF), Translational Health Science and Technology Institute, NCR Biotech Science Cluster, Faridabad, India, registration number: 1685/GO/ReBi/S/2013/CPCSEA.

### Animal Immunization

For animal immunization studies, 7-8-week-old C57BL/6 (male) mice bred in the THSTI small animal facility (SAF) were used; five animals per group were immunized with different formulations following the prime/boost immunization regimen as mentioned in **Figure 4A**. Each experimental group of animals was immunized three times with 30 µg of purified RBD at 21 days apart in different adjuvant and non-adjuvant formulations (at 1:1 ratio) except the animals in control groups. The control groups (three in number; PBS, AddaVax, Imject) were immunized with the same volume of PBS as used in the experimental groups along with adjuvants. The PBS control group received 100 μl of PBS (50 μl of PBS+50 μl of PBS), the AddaVax group received 50 μl of PSB mixed with 50 μl of AddaVax and Imject group animals were immunized with 50 μl of PBS mixed with 50 μl of Imject. The RBD protein (30 µg) was mixed with PBS for the antigen alone group and with AddaVax before immunization, while for the Imject group the antigen (RBD) was adsorbed with the adjuvant in a larger volume (at 1:1 ratio) overnight at 4°C before immunization. The animals were bled two weeks after each immunization. The sera were collected and stored at -80°C for future use.

### Anti-RBD Serum ELISA

The binding-antibody response to RBD and S2P proteins was measured using ELISA as described earlier (26). Serially diluted antisera (1:3 times) with 1:500 as a starting dilution in dilution buffer (1:5 times dilution of blocking buffer) were added to plate wells. The plates were incubated at room temperature (RT) for 1 h. The plates were then washed four times with washing buffer (PBS + 0.1% tween 20) and incubated with peroxidase AffiniPure goat anti-mouse IgG (1:2000 dilutions, Jackson Immuno Research, USA) for 45 min. They were subsequently washed with washing buffer four times, and then 100 ul of TMB substrate (Thermo Fisher Scientific) was added to the washed wells. The reaction was stopped by adding 100 µl of 1 N H_2_SO_4_ and the plates were read at 450 nm on a 96-well microtiter plate reader.

### *In Vitro* Stimulation of Splenocytes

Stimulation of splenocytes was carried out in a 96-well plate *in vitro* in the presence of PMA (20 ng/ml) and ionomycin (1 µg/ml) at 37°C for 6 h or protein antigen RBD or the spike protein (50 µg/ml) at 37°C for 72 h in a 5% CO_2_ incubator. Thereafter, the cells were pelleted down into 96-well plates and the culture soup were aspirated and used further for the quantitation of cytokines through ELISA. The stimulated cells were used for intracellular cytokine staining as mentioned below.

### Intracellular Cytokine Staining

Splenocytes stimulated *in vitro* by respective peptides or PMA+Ionomycin were stained for surface CD4-PerCp and CD8-FITC markers in the dark for 20 min at room temperature (RT). The cells were then permeabilized and fixed with BD Cytofix/Cyto Perm according to the manufacturer’s manual. Thereafter, IFNγ-Alexa Fluor 647, IL-17A-PE-Cy7, and IL-2-PE staining was carried out in Cytofix buffer at RT for 20 min in the dark. The stained cells were then washed and acquired on BD FACS Canto II and were analyzed on FlowJo software (Tree star).

### Cytokine ELISA

IFNγ, IL-17A, and IL-10 cytokines secreted in the *in vitro* stimulated culture soup were quantitated through sandwich ELISA by using anti-mouse IFNγ, IL-17A, or IL-10 primary and secondary antibodies. Briefly, the primary antibody was coated overnight and then washed and blocked with 5% BSA solution at 37°C for 4 h. The culture soup was then added to each well (1:1 dilution) and incubated at room temperature (RT) for 2 h, followed by 1 h incubation with biotinylated secondary antibody. Combination of HRP-Peroxidase and TMB substrate followed by 0.2 N of HNO3 stop solution was then used to develop the final color. The change in color intensity was then measured by using a spectrophotometer at 450 nm.

### IgG Purification

Pooled sera (equal volume from each animal) from each immunized group (control and experimental) were diluted 20-fold in PBS and incubated with protein G resin for 2 h at room temperature. The resin-sera post incubation was spun at 3,000 rpm in a swing out rotor for 5 min. The supernatant was removed from the tubes and resin was washed three times with PBS and one time with phosphate buffer with 500 mM of NaCl. The IgG was eluted from the resin by 0.1 M of glycine (pH 2.8), the eluted IgG was immediately neutralized with 100 mM of Tris (pH 8.0). Purified IgG was further dialyzed and concentrated prior to BLI and immunofluorescences studies.

### Serum IgG-ACE2 Competition Using Biolayer Interferometry BLI

The anti-RBD serum IgG and ACE2 competetion assay was performed using the Octet Red96 instrument (ForteBio, Inc., USA). For each set of experiments, 5 AHC sensors were used, in 4 out of 5 sensors RBD-Fc was captured at a 10 µg/ml concentration for 200 s. The one uncaptured sensor was used to check the background binding interaction of mouse IgG to the anti human Fc capture sensors and not used further in the experiment. Two of the RBD-Fc capture sensors were then incubated with purified IgG for 800 s to reach RBD and anti RBD-IgG saturation. The other two RBD-Fc capture sensors were not incubated with the purified IgG. For the determination of ACE2 binding inhibition, one sensor from each set (RBD+Anti RBD IgG and RBD+no IgG) was incubated with ACE2-his. The binding response of RBD-Fc to ACE2 was normalized to estimate the inhibition in ACE2 binding on the sensors where RBD-Fc was incubated with anti-RBD IgGs. The whole set of experiments was performed individually for each immunized group and repeated three times (the Imject + RBD set was repeated five times).

### Competitive Inhibition ELISA Assay

MaxiSorp ELISA 96-well plates were coated with ACE2-His protein in coating buffer overnight, blocked for 1 h with 3% skim milk prepared in PBST. RBD-Fc was incubated for 1 h at room temperature with a serial dilution of immunized mice pooled sera from the RBD immunized and control group. The above mixture post incubation was added to the ACE2-coated blocked plates and incubated further for 1 h at RT. After three washes with PBST, HRP-conjugated anti-human Fc was added as a secondary antibody and incubated for 1 h at RT. The following procedures were the same as described in ELISA.

### Immunofluorescence Microscopy

Immunofluorescence staining was performed to detect the binding interaction between anti RBD sera IgG with cell line HUH7 expressing the SARS-CoV-2 spike protein. Briefly, HUH7 cells were seeded in glass coverslips at 50-60% confluency with 10% FBS-supplemented DMEM media in a 12-well plate. One µg of pCMV14-3X-Flag-SARS-CoV-2 S plasmid was transfected in each well using lipofectamine 2000. Six h post transfection the old media was replaced with fresh media and incubated for the next 36 h. After 42 h, the transfection cells were washed with 1XPBS followed by fixation with 1 ml of 100% chilled methanol for 5 min in -20°C and washed three times with 1XPBS. Cells on the coverslip incubated with blocking solution (3% BSA in 1XPBS) for 1 h at RT. Next, coverslips were washed once with 1XPBS and incubated with anti-flag and anti RBD sera (from each immunized group and control group (equal concentration of sera from each group diluted in 1:50 ratio in 1XPBS+3%BSA)) for 1 h at RT. For control, one coverslip with spike-expressing cells was incubated with blocking solution only for 1 h at RT. After 1 h of incubation, the coverslips were washed with 1XPBS three times for a minimum of 5 min each wash followed by secondary antibody staining using anti-mouse Alexa Fluor 488 and goat anti flag Alexa Fluor 647 (Thermo Fisher Scientific, Invitrogen, USA) with 1:500 dilution for 1 h at RT. After secondary antibody incubation, the coverslips were washed with 1XPBS three times. Next, the coverslips were mounted on a glass slide using ProLong Gold (anti-fade reagent, Invitrogen, USA) containing DAPI (4’, 6’-diamino 2-phenyl indole). Images were acquired on an Olympus FV3000 confocal microscope with a 60X (NA 1.4) objective.

### FACS Analysis With the HEK293T Cells

Two million HEK293T cells were seeded in a 100 mm cell culture petri-dish the day before transfection. Twelve ug of the plasmid (pCMV14-3X-Flag-SARS-CoV-2 S) was transfected with a 1:1 ratio of lipofectamine 2000 (Thermo Fisher scientific). After 48 h, the cells were gently washed with 1xdPBS and were harvested in FACS buffer (1xPBS + 10%FBS +1mM EDTA). Cells were washed in FACS buffer two to three times with gentle centrifugation (250 g) for 5 min at RT. Cells were resuspended in 3 ml of FACS buffer and the 0.1-0.2x10^6^ cells were aliquoted in a 1.5 ml microcentrifuge as per the requirement. Each tube was incubated with 20 ug/ml of primary antibody (pooled mouse serum IgG from anti-RBD, anti-RBD+AddaVax, anti-RBD+imject, and control group was used as primary antibody) for 1 h at RT followed by washing with FACS buffer three times; here untransfected cells were also used as the untransfected mock control. Further cells were incubated with florescence dye conjugated secondary antibody (Alexa Fluor 488) at 1:400 dilution, followed by washing with FACS buffer and fixing with 4% PFA for 10 min at RT. Samples were acquired in BD FACSCanto II and raw data were analyzed using FlowJo software.

### Plaque Reduction Neutralization Tests (PRNT) Assay

A total of 0.1 million Vero-E6 cells were seeded into each well of a 24-well plate, at least 20 h before the experiment. Serum samples were heat-inactivated at 56°C for 1 h and subsequently serially diluted two-fold using DMEM supplemented with 2% FCS. SARS-CoV-2 virus stock was diluted to produce 30 plaque-forming units (Pfu) per well. Virus dilution was mixed with serial dilutions of sera and incubated at 37°C, for 1 h. Subsequently the mix of serum and virus were overlaid on a VeroE6 monolayer and the plates were incubated for 1 h at 37°C in 5% CO2 with intermittent rocking. At the end of incubation, the inoculum was discarded; the monolayer in each well was washed with serum-free media and DMEM-2%-FCS supplemented with 1.5% carboxymethyl cellulose was overlaid onto the monolayer. The plates were incubated for 72 h in 37°C and 5% CO_2_. The monolayer was fixed using 4% PFA and the plaques were visualized by crystal violet staining. The number of plaques in the wells with no sera were counted and taken as control. The average number of plaques corresponding to each serum dilution was expressed as a percentage of that in the control wells. The inverse value of the dilution that reduced the plaque numbers to 90% of that in the control wells was taken as PRNT_90_.

### Virus Neutralization Assay

A serum neutralization test was carried out to determine the presence of neutralizing antibodies in the immunized serum samples. All the serum samples were heat-inactivated at 56°C for 1 h before testing with the SARS-CoV-2 virus at BSL3. The virus was obtained from BEI Resources (isolate USA-WA1/2020) and passaged in Vero E6 cells, titrated, and 1 x 10^2^ TCID_50_ of virus (diluted in 50 µl of the serum-free media) was incubated with 50 ul of serially two-fold-diluted serum samples from a starting dilution of 1:20 for 60 min at 37°C. The virus-serum mixture was transferred to Vero E6 cells and allowed to adsorb/infect for 1 h at 37°C with 5% CO_2_. Vero E6 cells were subsequently washed with serum-free media and DMEM media was added which was supplemented with 2% FCS. Cells were incubated for 72 h days at 37°C with 5% CO_2_ and the presence of CPE were observed under a microscope daily.

### Statistics

All statistical analysis was performed using GraphPad Prism 7 software. Sera end point titers were calculated as the reciprocal of serum dilution giving O.D 450 nm readings higher than the lowest dilution of the placebo or control arm + two times standard deviations. No samples or animals were excluded from the analysis, pooled sera was prepared with an equal volume of serum from each individual animal in the group.

## Results

### Identification of Receptor Binding Domain of SARS-CoV-2

The RBD of coronaviruses has been shown to fold independently upon expression and elicits a robust neutralizing antibody response (27). In order to deduce the sequence coding for the RBD, we analyzed the SARS-CoV-2 sequence (GenBank: MN988668.1) (28). Structure-based sequence alignment employing PDB blast (Blastp with PDB database) was used to identify the structures with maximum identity. Three homologous hits; 2GHV (18), 2DD8 (19), and 3BGF (29), were identified with 73.60%, 74.11%, and 73.71% of identities, respectively (**Figure S1**).

**Figure 1:**
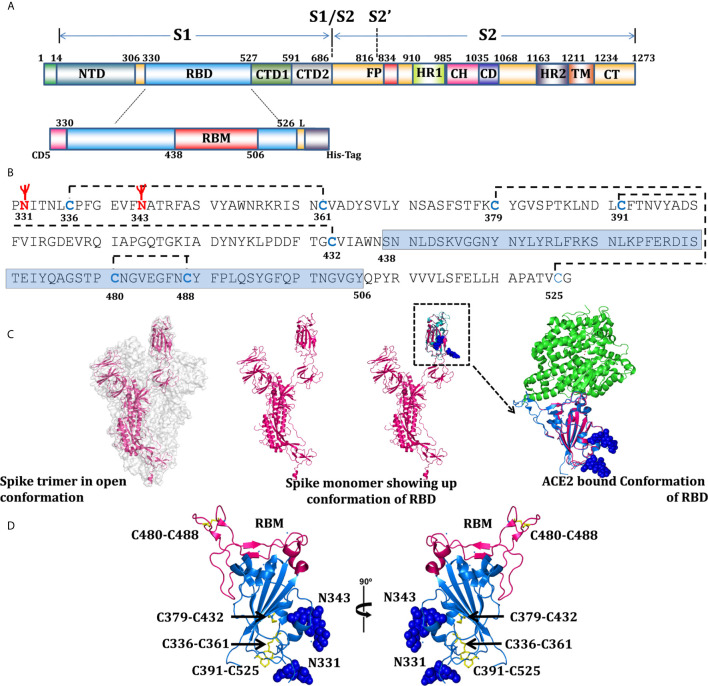
Domain organization of SARS-CoV-2, RBD expression construct, and overall structural topology. **(A)** The schematic presentation of domain organization for the SARS-CoV-2 spike protein, differentially colored boxes represent domain boundaries; NTD (N-terminal domain), RBD (receptor binding domain), CTD1 and 2 (C-terminal domain 1 and 2), FP (fusion peptide), HR1 (heptad repeat 1), CH (central helix), CD (connector domain), HR2 (heptad repeat 2), TM (transmembrane domain), and CT (C-terminal domain) (30). The schematic representation of RBD; with N-terminal CD5 secretary sequences, coding sequences (330-526), RBM (receptor binding motif), 4 amino acid linkers, and 6x histidine tags. **(B)** Analysis of RBD coding sequences showing probable glycosylation sites at N331 and N343, cysteine residues - disulfide bonding network and sequences coding for RBM. **(C)** Surface representation of spike trimer in open conformation (6VYB) (31) with one monomeric subunit shown in the cartoon presentation to represent the overall organization of a monomer in the trimeric spike structure. One monomer of the trimer superimposed with RBD structure (6VW1) (32) and inset showing RBD in ACE2-bound conformation, showing the potential glycan in sphere representations. Residues 330-334 represented through modeled and superimposed structure (pink), showing the probable position of glycan at N331. **(D)** Overall structure of RBD in two orientations, RBD’s (blue), RBM shown in light pink color, cysteine residue forming disulfide bonds drawn as sticks and shown in yellow color, and two glycans are shown with sphere representation.

In order to maintain proper folded conformation with paired cysteine residues (**Figure 1B**), ACE2 binding sites (**Figure 1C**), and natural N-linked glycosylation sites (**Figure 1B**), we selected _330_PNITNLCPF to HAPATVCG_526_ amino acid sequences of the SARS-CoV-2 spike protein to express as the RBD of SARS-CoV-2 (**Figure 1A**). The selected sequence RBD_330-526_ (henceforth designated as RBD) has eight cysteine residues (**Figures 1B, D**) and two potential N-linked glycosylation sites at amino acids N331 and N343 (**Figures 1B, D**) and a receptor binding motif (RBM) from 438-506 (**Figure 1**). Since glycosylation likely plays a role in protein folding and immune evasion, we designed our expression construct RBD with native glycosylation sites N331 and N343, which were expressed and purified through the mammalian expression system, to be tested as a potential vaccine candidate.

### Characterization of SARS-CoV-2 RBD

**Figure 2:**
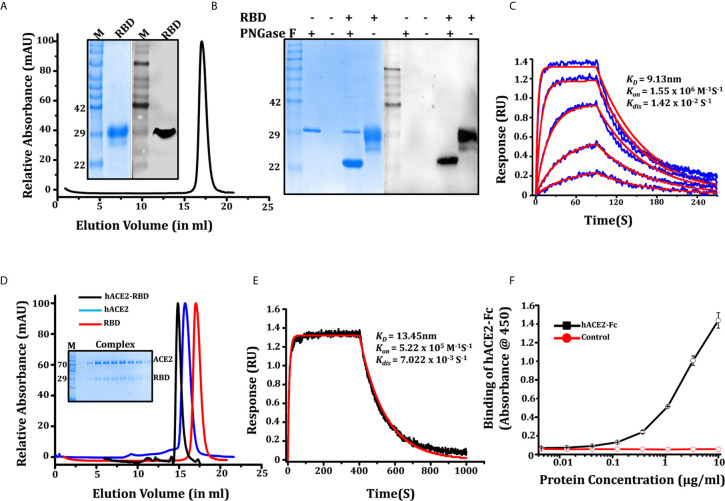
Characterization of SARS-CoV-2 RBD. **(A)** Purification of RBD through SEC, protein elutes as single oligomeric population with peak position at 17.04 ml, inset showing SDS PAGE and western blot (anti-his antibody) of purified RBD recognizing a 32 kDa band. **(B)** SDS-PAGE and western blot showing the glycosylation status of the RBD, western blot confirming the glycosylated and de-glycosylated conformation of RBD. **(C)** Binding kinetic evaluation of mAb CR3022 to RBD at nanomolar affinity, the RBD used as analyte in various concentrations (shown in blue color graphs). The red solid line shows the fitted curve. **(D)** Gel elution profile of RBD, hACE2-His, and RBD-hACE2 complex shows a shift in the peak of the complex toward higher molecular weight oligomeric conformation, SDS gel in inset showing the co-elution of RBD and hACE2 as a complex. **(E)** Binding of biotinylated hACE2 with RBD on BLI platform, our result shows that ACE2 binds efficiently to RBD with K_D_ of 13.43 ± 0.80nM. **(F)** Dose-dependent binding response of hACE2-Fc with RBD measure with ELISA.

The gene sequence coding for RBD was codon-optimized for expression in the mammalian expression system and was cloned with N-terminal secretory sequences and C-terminal histidine tags (**Figure 1A**). The expressed supernatant post transfection was purified using Ni-NTA affinity purification followed by gel filtration chromatography. The size exclusion chromatogram confirmed the protein as a monomer. The molecular weight for RBD as analyzed by Coomassie brilliant blue staining on SDS PAGE comes out to be 32kDa (**Figure 2A**), with 95% purity and a total yield of 32 ± 8mg/l. Since the calculated molar mass as per the amino acid composition of the RBD expression construct was 23.4kDa, we explored its glycosylation status by deglycosylation using deglycosylating enzymes PNGase F and endoglycosidase H, followed by a comparison of migration. As observed earlier in SARS-CoV, where a mutation of each N-linked glycosylation site alanine or glutamine resulted in a decrease of the relative molecular weight by approximately 3kDa (27), we found that complete deglycosylation of RBD with PNGase F, which cleaves the GlcNAc on asparagine residues by hydrolyzing the amide side chain of the asparagine under a native condition, reduced the molecular weight by approximately ∼6-7kDa. Both PNGase F (**Figure 2B**, **Figure S2A**) and endo H de-glycosylases increased the relative migration, suggesting the presence of two potential glycosylation sites in the protein. Peptide mass finger printing of purified protein following trypsin digestion confirmed that the peptide fragments correspond to the SARS-CoV-2 spike protein receptor binding domain with 35% of the total sequence coverage (**Figure S2C**).

We further characterized the potential of RBD to interact with hACE2 (human ACE2, Supporting information) using the monoclonal antibody CR3022 and anti-SARS polyclonal antibody (Anti SARS-pAb). Four amino acid changes in the ACE2 binding ridge of the SARS-CoV-2 RBM (482-485;Gly-Val-Glu-Gly) makes the structure more compact and facilitates the accessibility of the RBD to the N-terminal helix of ACE2. Therefore, we studied the hACE2-RBD interaction in solution to form a complex. RBD and hACE2 (mixed in 1:2 molar ratios) elutes a complex from the size exclusion column. The elution profile shows a shift in peak toward higher molecular weight, and SDS PAGE suggests co-elution of the proteins as a complex (**Figure 2D**). We next tested biotinylated hACE2 (Supporting information: hACE2 biotinylation) binding with RBD using a BLI platform. We found that hACE2 binds efficiently to RBD; K_D_ of 13.43 ± 0.80nM, with *Kon* 5.2 x 10^5^ (1/Ms) and *Kdis* 7.022 x 10^3^ (1/s) (**Figure 2E**), which is in agreement with recently published parameters (15). We also accessed the binding of hACE2-Fc (Supporting information, **Figure S3A**) to RBD which binds with K_D_ of 11.17 ± 0.62 nM (**Figure S3B**). A similar assessment on an ELISA platform showed that ACE2-Fc binds to RBD with EC_50_ of 2.015 ug/ml (**Figure 2F**). The specificity of RBD to interact with mAbCR3022 and Anti-SARS pAb was measured using ELISA. As expected, RBD recognized and interacted strongly with mAb CR3022 and anti-SARS-CoV-2 polyclonal antibody (**Figures S2D, E**). The western blot performed using anti-his antibody (**Figure 2A**) and anti-SARS-CoV-2 polyclonal antibody (**Figure S2B**) recognized the 32KDa band of the purified protein. The bio-layer interferometry (BLI) performed using monoclonal antibody CR3022 shows its binding to RBD with K_D_ of 9.13 ± 0.91nM (**Figure 2C**).

### Stability and Prolonged Survival of RBD Vaccine Candidate, Estimation Over Temperature Range and Storage Condition

**Figure 3:**
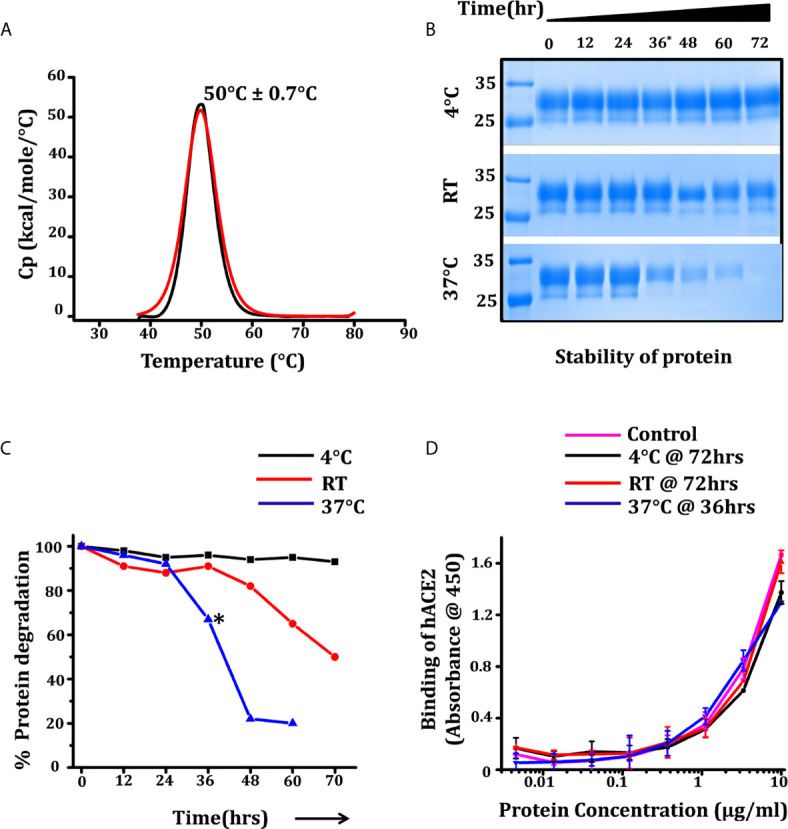
Thermostability and prolonged survival of RBD over temperature range. **(A)** Differential scanning calorimetry (DSC) measuring the melting temperature (*T_m_*) of RBD, black and red graphs showing the thermogram and fitted curve. **(B)** SDS protein gel showing the stability of protein for 72 h at three different temperatures, each aliquot was harvested at 12 h intervals. **(C)** Density intensity plot of RBD-incubated samples were analyzed using ChemiDoc MP (version 4.1) and plotted with reference to the samples intensity at 0 h. **(D)** ELISA graph showing the dose-dependent reactivity of end point incubated RBD (72 h for 4°C and RT incubation and 36 h for incubated samples at 37°C) with hACE2-Fc, the control graph represents the relative activity of RBD at the 0 h time point. Statistical significance was determined using the one-way ANOVA test (p < 0.05). Where *p < 0.05 (One-way ANOVA).

Thermo-stability and thermo-tolerance are two important parameters for a successful vaccine candidate. We estimated the thermo-stability of RBD by differential scanning calorimetry (DSC), measuring the melting temperature (*T_m_*) and thermo-tolerance of the purified RBD at different temperatures for 72 h. The *T_m_* for RBD as estimated through DSC was 50°C ± 0.7°C with onset of melting at 42°C (**Figure 3A**). The thermo-tolerance parameter which is associated with prolonged survival and storage as well as with the shipment of the vaccine (ambient to extreme condition (cold chain or -20/-80°C)) was measured by incubating the protein at different temperatures; 4°C, room temperature (RT) (24-25°C), and at 37°C. After every 12 h of incubation the protein was analyzed for its efficacy to interact with monoclonal antibody CR3022 (cryptic epitope) and the ACE2 receptor. We observed that at 4°C there was no obvious protein degradation or loss of activity up to 72 h of incubation, whereas at RT, no significant degradation was observed up to 48 h of incubation. However, at 37°C we observed a 40% loss of protein at 36 h of incubation (**Figures 3B, C**) although the potency of the incubated protein post incubation to interact with the ACE2 receptor remained unchanged (**Figure 3D**). No degradation or loss of activity was observed in protein stored at -20°C and -80°C following a repeated freeze thaw cycle over a period of three months, suggesting the RBD displayed vaccine candidate suitability and sustainability as vaccine targets and toward storage and transportation condition.

### SARS-CoV-2 RBD-Specific Humoral Immune Responses in Immunized Mice

**Figure 4:**
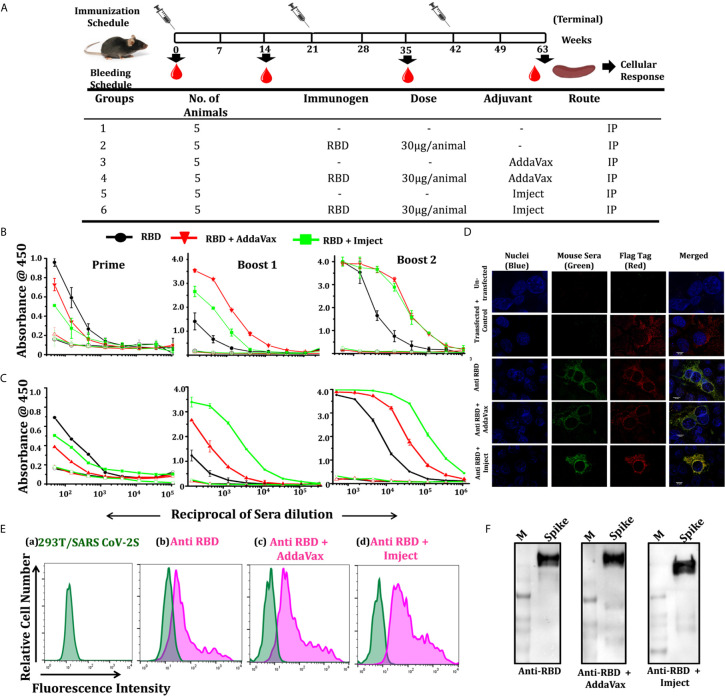
Immunization strategy, RBD specific antibody titers, and cell surface interaction with spike. **(A)** Schematic representation of the immunization schedule of mice, showing immunization and bleeding time point, different groups, number of animal/group, immunogen, immunogen concentration, used adjuvants, and route of administration. Humoral response studies at each bleeding time point and cellular response studied 14 days post final immunization. **(B)** Dose-dependent binding response of RBD-immunized sera in different formulation towards RBD and pre-fusion spike trimer (S2P) (expression and purification spike trimers elaborated in **Supplementary Information**). **(C)** Prime; starting dilution used was 1:50, followed by 3-fold serial dilution of sera, boost 1; starting dilution used was 1:150, shows no significant increase in antibody titer in RBD alone group post first boost, with increase in binding antibody response in the other groups, boost 2; increased of antibody titers in all the RBD immunized groups with the RBD + AddaVax and RBD + Imject groups showing high end point titers (starting dilution 1:500). The overall response toward the spike trimer was similar to the RBD with a higher end point titer as compared to RBD. **(D)** Immunofluorescence detection of SARS-CoV-2 spike (SARS-CoV-2 S with 3X Flag-tag) by RBD immunized sera: immunized serum IgG from different immunized groups was used as a primary antibody and probed with secondary antibody Alexa-Fluor 488-labeled anti-mouse antibody (green) (1:100). Anti-Flag antibody (red) and nuclei were stained with 4’,6-diamidino-2-phenylindole (DAPI, blue). Scale bar: 10 μm and magnification 60x. **(E)** Representative overlaid histograms of SARS-CoV-2 S with 3X Flag-tag transfected 293T cells, a) SARS-CoV-2 S transfected 293T cells, b) stained with anti-RBD IgG, c) stained with anti-RBD+AddaVax IgG, and d) stained with anti-RBD+Imject IgG as the primary antibody. Anti-RBD antibody binds strongly to the SARS-CoV-2 spike expressed at the cell surface, **(F)** western blots using pooled sera from each RBD formulation immunized group was used as a primary antibody, which efficiently recognizes a spike protein band of ∼180 kDa.

To study the immune response of the SARS-CoV-2 RBD in a comparative and unbiased manner, we selected C57BL/6 mice for assessment of immunogenicity and compared the potency of different formulations (adjuvant versus non-adjuvant combinations, to mimic prophylactic vaccination and natural infection, respectively). As both humoral and cell-mediated immune responses are expected from a potential vaccine candidate, we selected here the intraperitoneal (i.p.) route for immunization. The i.p. route of administration (due to the mesenteric lymph node in the peritoneal cavity) has a better chance of activating the naïve T cells which recognize the antigens to generate a robust response (33, 34). Therefore, C57BL/6 male mice, in groups of five animals/group, were intraperitoneally (i.p.) immunized with RBD as an antigen either without any adjuvant (in combination with saline) or in formulation with AddaVax or Imject as the adjuvant. Animals in three additional control groups received: PBS, PBS + AddaVax and PBS + Imject and PBS formulation (**Figure 4A**).

AddaVax^(R)^ is a squalene-based oil-in-water nano-emulsion (similar to MF59) which can enhance both cellular and humoral immune responses (35) and has been approved to boost efficacy of seasonal influenza vaccine. Imject on the other hand is an alum-based formulation with aluminum hydroxide and magnesium hydroxide. Alum or alum-based salt compositions have been in use for human vaccines since the 1930s and are known to promote a Th2 type bias to immune responses (36). We used a prime/boost immunization regimen where we primed the animals at day 0 and boosted the immune response with different adjuvant/non-adjuvant formulations at 21 days apart. We collected the pre-bleed sera before the start of immunization and post-immunization bleeds were collected 14 days after each dosing (**Figure 4A**). Following immunization, we compared the response of the immunized animals initially from pooled sera from the respective group followed by individual animal serum against RBD and pre-fusion ectodomain spike trimer protein (response toward spike ectodomain). We initially estimated the whole IgG response of the sera collected after prime, followed by boost 1 and boost 2 through antigen ELISA (RBD, pre-fusion spike trimers). Other than the control groups (PBS alone, PBS + AddaVax and PBS + Imject), all other groups which were immunized with the antigen (RBD) formulation showed a very high RBD- and spike-specific immune response, including the group which was immunized with RBD alone (without any adjuvant).

Pooled sera from the RBD non-adjuvanted group and RBD + Imject groups showed detectable RBD-specific antibody titers 14 days after the first immunization (prime). The response evaluated after boost 1 showed a sharp rise in RBD-specific antibody titers in the AddaVax+RBD group, followed by the Imject+RBD group. However, with this boost dose not much increase in the titer was seen in the antigen alone group (**Figure 4B**). When immune response was evaluated 14 days post third immunization (boost 2), RBD-specific responses were the same for all three antigen formulation-immunized groups. No significant detectable antibody titer was observed in any of the control groups.

We further enumerated the reactivity and response of RBD-immunized animal sera toward pre-fusion spike trimer (**Figure S5A**) using trimeric spike protein-coated ELISA (**Figure 4C**). The overall end point titer for the RBD immunization post 2^nd^ boost at different formulations was investigated and found to be very high; 1:121500 for the RBD alone group when tested against self-antigen (**Figure 4B**) as well as the spike trimer (**Figure 4C**). For the formulation with AddaVax and Imject the end point titer was found to be in the range of 1:364500 toward RBD (**Figure 4B**) and higher than 1:1093500 when tested against the spike pre-fusion trimer (**Figure 4C**).

We further analyzed the ability of the immunized pooled sera to bind selectively to the spike protein, expressed on cell surface, and with the pre-fusion soluble spike (S2P) protein, through immunofluorescence staining, western blot, and BLI studies. The binding specificity of the pooled sera from each immunized group was determined by indirect immunofluorescence and *via* cell surface interaction to SARS-CoV-2 S (with 3X Flag-tag at the C-terminal). Sera from the immunized group recognized the expressed spike trimer and co-localized with the flag tag (**Figure 4D**), the results from immunofluorescence staining revealed positive signals for sera from the antigen immunized groups (**Figure 4E**). The western blot shows that pooled sera from the immunized group efficiently recognized the soluble pre-fusion spike trimer (**Figure 4F**). The purified IgG (of the RBD-immunized group) binds to the spike trimer in BLI-based studies (**Figure S5B**). We also analyzed the serum binding reactivity of RBD-immunized individual mice in each group (five animals in each group) to identify any outlier and significant contributions from all animals in the group response (**Figure S5D**).

### IgG Subtyping of Non-Adjuvant and Adjuvant Formulation Groups

**Figure 5:**
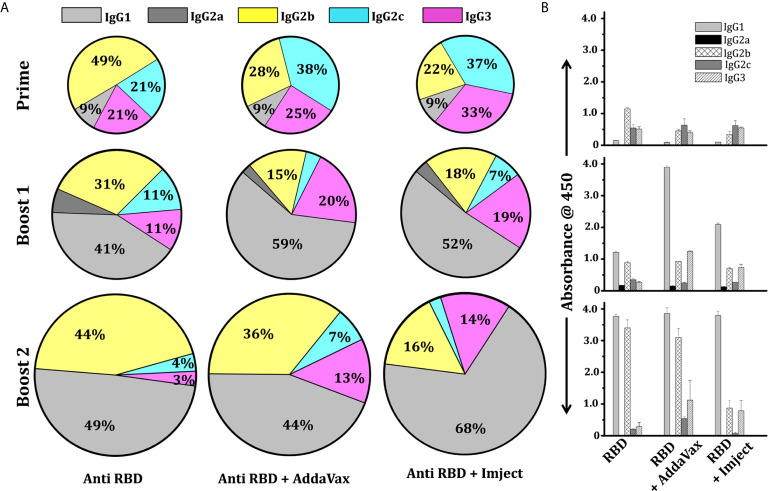
IgG subtyping of adjuvant and non-adjuvant group. **(A)** Pie chart shows the mean percentile of serum IgG subtype feature of different RBD immunized groups at different time points. The colors represent the type of IgG response; grey, dark grey, yellow, cyan, and pink designate IgG1, IgG2a, IgG2b, IgG2c, and IgG3. The marked percentage in each subset designates the contribution of each subclass in the overall response. **(B)** Binding IgG subtype titer response showing the mean response of serum IgG subtype feature of different immunized groups at different time points with standard deviation.

In the mouse model, the IgG subclass induced after immunization could reflect the nature of the antigen. Therefore, we further enumerated the IgG subclass response of immunized mice immunized with different formulations to determine Th1/Th2 polarization. We observed that the overall response was dominated by the IgG1 subclass in all immunized groups (**Figure 5A**). In the RBD alone group (i.e., without any adjuvant), following priming (first immunization), the response was predominated by the IgG2b subclass which switched to and was subsequently dominated by the IgG1 subclass after the second and third immunization (boost 1 and boost 2) (**Figure 5A**). However, a very prominent IgG1 antibody titer was seen in both adjuvant groups (AddaVax and Imject group) post second immunization (boost 1). The IgG2 level was comparable among the RBD alone and RBD + AddaVax groups, the latter group along with the RBD+Imject group also showed a significant amount of the IgG3 subclass (**Figures 5A, B**). The animals immunized with RBD + Imject showed a significantly dominant IgG1 subclass response. After third immunization (boost 2), all the three immunized group responses were dominated by the IgG1 subclass followed by IgG2b and IgG3. The IgG2c response contribution was negligible in RBD+Imject sera post second immunization (boost 2) (**Figures 5A, B**). However, how this class and subclass of antibodies are related to cellular response for clearance of viral load post infection, or have the potency and efficacy toward virus neutralization, or the potential to inhibit the ACE2 receptor and RBD interactions, need further investigation.

### Assessment of Cellular Immune Responses Post RBD Immunization

**Figure 6:**
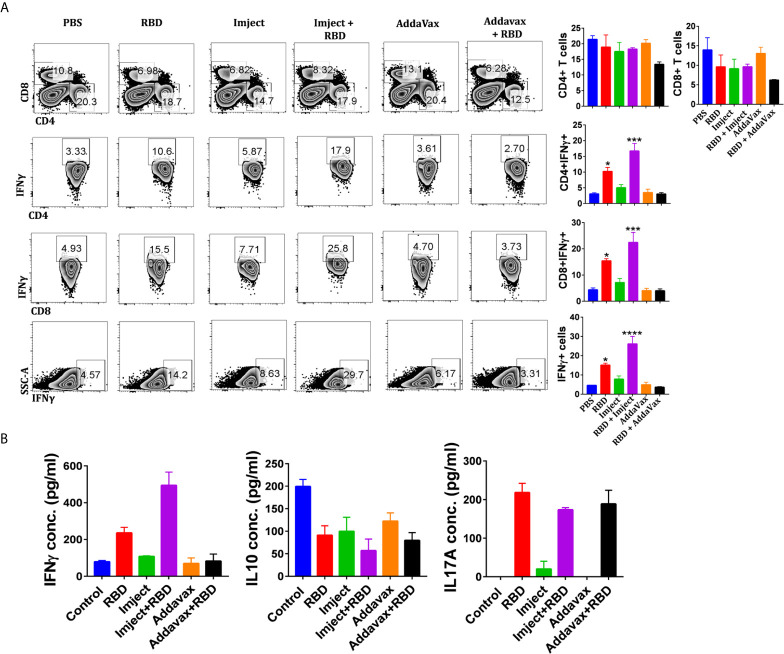
Assessment of antigen-specific cellular immune responses. **(A)** Splenocytes harvested 14 days post second boost were used for the T cell response studies. *In vitro* antigen-specific (RBD) stimulated splenocytes were used for intracellular cytokine staining of IFNγ, IL-2, and IL-17A cytokines after CD4 and CD8 surface staining. Stained cells were acquired on BD FACSCanto II and analyzed on FlowJo. **(A)** The contour plots (left panel) indicating mean percent positive values for various T cell populations in the antigen-stimulated splenocytes. Representative bar graph (left panel) plotted for percent of positive ± standard errors of the mean (SEMs) for each group. **(B)** Sandwich ELISA of IFNγ, IL-17A, and IL-10 cytokines of the *in vitro* RBD-stimulated culture soup was quantitated by using anti-mouse IFNγ, IL-17A, or IL-10 primary and secondary antibodies. Statistical significance was determined using the one-way ANOVA test (p < 0.05). where *p < 0.05, ***p < 0.001, and ****p < 0.0001 (one-way ANOVA).

As neutralizing antibodies could prevent viral entry, the potential response toward coronaviruses requires an antigen-specific T cell response (37). CD4+ T helper cells are required for the generation of neutralizing antibodies; antigen-specific CD8+ T cells are critical for eliminating virally infected cells. Depletion of CD4+ T cells has been shown to delay the virus clearance and enhance immune-mediated interstitial pneumonitis and reduce a neutralizing titer in the lungs of SARS-CoV-infected mice (38). Similarly, CD8+ cytotoxic T cells play a crucial role in recognizing and killing the infected cells (39). In the absence of CD4+ T cells, CD8+ T cells have been shown to provide a partial but significant level of protection and virus clearance (37). Hence, we investigated the T cell-mediated immune response of the SARS-CoV-2 RBD antigen in mice immunized with adjuvant and non-adjuvant formulations. The splenocytes were harvested 14 days after boost 2 and re-stimulated *in vitro* with viral proteins RBD (**Figures 6A, B**) and PMA/ionomycin (**Figures S6A, B**). We observed that the Imject + RBD group induced significant upregulation in the frequency of IFN-γ secreting T helper cells as well as cytotoxic T cells across all stimulation conditions (**Figures 6A, B** and **Figures S6A, B**). IFN-γ directly inhibits viral replication and enhances the antigen presentation (40). Interestingly, the induction of IFN-γ was found to be 2-fold or higher in groups immunized with Imject + RBD as compared to RBD alone suggesting that alum formulation might be helping the priming of the T cell immune response. The intracellular cytokine profile also corroborated with an increase in the secreted IFN-γ levels in ELISA as there was a 2-fold increase in secreted IFN-γ concentration in the Imject + Antigen vaccinated group in comparison to the RBD alone group (**Figure 6B**), and it was 5-fold higher in comparison to AddaVax + RBD (**Figure 6B**). Since a robust T cell-based IFN-γ (a marker of Th1 response) (41, 42) production is important for mounting a potent anti-viral response, we speculate that the Imject + RBD formulation could provide insight for a potential formulation in RBD-based vaccine development. Further, we also investigated the level of IL-2 secretion upon PMA/Ionomycin stimulation (**Figures S6A, B**). Surprisingly, we did not found any significant difference in the induction of IL-2 between the RBD alone and other RBD formulation groups (**Figure S6A**). It must be noted that, even though non-significant, we observed some basal levels of elevated IL-2 secretion in all the immunized groups.

We also investigated the role of IL-10, a mediator of anti-inflammatory response which works as an immunosuppressant against a wide range of cytokines and chemokines. Its secretion was inhibited (**Figure 6B** and **Figure S6B**) across all RBD immunized formulations as compared to control groups, which is very important here to mount a strong anti-viral IFN-γ response as evident from our cytokine data.

### Pathological Correlation of Different Immunized Groups

Recent studies have shown that host Th-17 inflammatory responses contribute toward severe lung pathology and mortality of lower respiratory tract infections from coronaviruses (43). Th-17 inflammatory responses in COVID-19 individuals are associated with the release of key cytokines including IL-17. Previous studies with RBD-based immunization rule out any antibody-dependent enhancement (43). Alum or alum-based adjuvants have been shown to be associated with immunopathological reduction and immune enhancement associated with IL-17 response in laboratory animals (43). Since there is no clarity about the T cell response in SARS-CoV-2 patients in terms of its protective or pathogenic nature, we therefore studied the changes in IL-17A secreting CD4 and CD8 T cell populations. Our PMA/Ionomycin stimulation data indicate that the RBD (RBD alone) immunized group elicited a 3-fold increase in CD4+Th17 cells and a 5-fold increase in the frequency of CD8+IL-17A T cells when compared to control groups (**Figures S6A, B**), suggesting that RBD protein may stimulate the pathogenic arm of adaptive immune response (**Figures S6A, B**). The AddaVax + RBD group showed a 2-fold enhancement in the frequency of both CD4+ and CD8+ Th-17 cells. The cytokine data also signify a similar trend of IL-17A expression among immunized groups (**Figure S6B**). Further, the Imject + RBD group, following antigen stimulation (**Figure 6B**), also showed a decrease in the frequency of IL-17 secretion when compared with the RBD alone and AddaVax+RBD groups. It is possible that the magnesium present in Imject inhibits macrophage activation through blocking certain calcium channels, contributing toward the anti-inflammatory effect which may divert the priming of pathogenic T cells toward protective T cell response (**Figures 6A, B**) (44).

### Inhibition of Binding to hACE2 Receptor Indicative of Neutralization Potential

**Figure 7:**
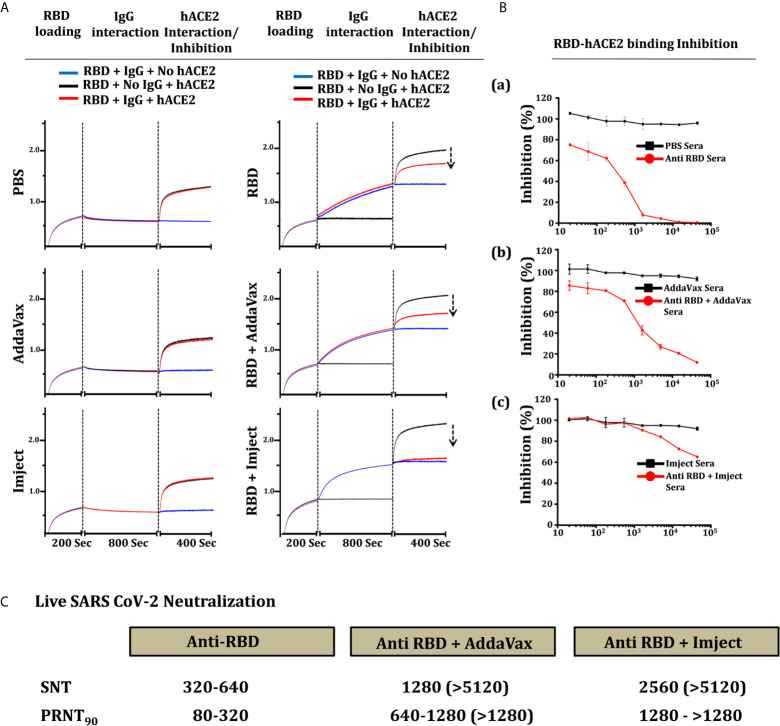
hACE2 competition, inhibition, and neutralization antibody titer estimation. **(A)** Competition of RBD-immunized sera IgG (right) and control group sera IgG (left) with hACE2 to bind to RBD. The section of vertical dashed lines indicates RBD-Fc loading, the start of the IgG association to RBD and complex formation, and free RBD and RBD-IgG complex solid-phase ACE2 interaction. The solid-phase hACE2 interaction was normalized to evaluate the reduction in RBD-IgG complex-hACE2 interaction. **(B)** Inhibition of RBD-Fc and hACE2 interaction in the presence of sera from respective control and RBD immunized groups. The percentage of RBD-hACE2 interaction was plotted with reference to control group sera reactivity, a) anti-RBD (RBD alone) sera, b) RBD + AssaVax sera and c) RBD + Imject sera. **(C)** Live virus neutralization of SARS-CoV-2 virus estimated through plaque reduction neutralization test_90_ (PRNT_90_) and serum neutralization test (SNT) of sera from each individual mouse from the group. The table shows the range of neutralization obtained from the mice in the group, the value bracket shows the highest response obtained from individual mice in the group.

We estimated the inhibition potential of SARS-CoV-2 RBD-immunized sera towards the hACE2 RBD binding inhibition through a biolayer informatory-based (BLI) platform and with a surrogate neutralization assay. Here we first loaded the purified RBD protein (RBD-Fc, **Supplementary Information**) to the BLI sensors, binding to the RBD-Fc (Fc from human IgG1 (45) with purified IgG from different immunized groups was tested and validated prior to the experiment (**Figure S7A**). Next, the RBD binds with purified pooled sera IgG were estimated to reach saturation. We further evaluated the binding potential of soluble purified hACE2 (ACE2-His) to RBD-sera IgG complex bound sensors and compared it with one without IgG (RBD-ACE2 interaction). The binding response of hACE2 in the groups with IgG from control groups (PBS, AddaVax, and Imject) matched exactly to the RBD-hACE2 interaction with no significant reduction in ACE2 binding suggesting that all ACE2 binding sites were unoccupied post control group IgG binding phase (**Figure 7A**). Further, we calculated the binding response of the antigen-based combination immunized group’s inhibition potential; the IgG from the RBD alone and RBD + AddaVax groups leads to a 32% and 49% reduction in ACE2 binding response respectively, suggesting some of the ACE2 binding sites on the RBD surface were preoccupied by ACE2-directed antibodies generated *via* RBD immunization. However, no binding of ACE2 was observed on sensors with a complex of RBD and IgG from the RBD + Imject group, showing that all the ACE2 binding sites were preoccupied by RBD+Imject IgG (**Figure 7A**). Here in this experimental setup, one sensor with no RBD was also used to rule out any background binding of mouse sera IgG’s with the experimental sensors (AHC anti human Fc sensors). The study implies the possibility of a potent ACE2-directed neutralizing antibody response from the Imject + RBD immunized group, which we further validated through ELISA-based RBD-ACE2 competition or a surrogate neutralization assay, or PRNT and SNT-based assays described below.

An RBD-ACE2 competition inhibition/surrogate assay was performed to estimate the binding potential of anti RBD and anti RBD + adjuvant sera toward ACE2 receptor protein. The inhibition in binding potential of RBD to hACE2 *via* RBD-immunized sera is used as an indicator for the presence of ACE2 binding site-directed neutralizing antibody response in the immunized sera. Anti RBD (RBD alone) sera inhibited the RBD-ACE2 interaction with IC_50_ of 380, the IC_50_ of RBD + AddaVax immunized sera was estimated as 1350 (**Figure 7B**). However, with the dilution used in the experimental setup being similar to the other immunized groups, only 40% inhibition was achieved which shows the presence of very high affinity ACE2-directed neutralizing antibodies in Imject+RBD immunized group animals (**Figure 7B**) and as an indication toward the whole virus neutralization potential.

### Neutralization Potential of SARS-CoV-2 RBD-Immunized Sera

To estimate the virus neutralization capacity of antibodies generated in response to the different immunization formulation, the serum collected from animals were tested by a serum neutralizing test (SNT) and a plaque-reduction neutralization test (PRNT) using replicative SARS-CoV-2 virus (SARS-related coronavirus 2, isolate USA-WAI/2020). Neutralization capacity (by SNT) was measured as the highest dilution of serum capable of preventing the development of the cytopathic effect (CPE) while in PRNT_90_ the dilution of serum which reduced the number of plaques by 90% when compared to no serum control was measured (PRNT_90_). Interestingly, RBD alone induced neutralizing antibodies that prevented CPE to a dilution ranging between1:320-1:640, as corroborated by PRNT as well as in the RBD-ACE2 competition assays (**Figure 7C**). This suggests RBD protein (RBD_330-526_) to be significantly immunogenic and even without any adjuvant, it can elicit a significant humoral as well as neutralizing antibody response. The RBD + AddaVax group showed the presence of a higher titer of neutralizing antibodies, showing PRNT90 values between dilution of 1:640 and 1:1280. Estimation of neutralization by SNT assay with sera from the same animals showed prevention of CPE formation even at a dilution of 1:5120 (**Figure S7B**). Similarly, the PRNT_90_ in sera from mice immunized with RBD-Imject was found to be higher than 1280 serum dilution, which was corroborated in SNT where no CPE was even observed at dilutions of 1:5120 of the serum (**Figure 7C** and **Figure S7C**). Taken together our data suggest that RBD, AddaVax, and Imject immunized groups showed the presence of neutralizing antibodies, with high neutralizing titer antibodies generated in the AddaVax and Imject groups (**Figure 7C**). The Imject + RBD-immunized group neutralizing antibody titer was highest than the other formulations studied in the present study, which was indicative of a potential protective response capable of controlling virus replication with no enhancement effect which is otherwise associated with suboptimal neutralizing antibody potency (46).

## Discussion

The unprecedented challenges posed by the novelty of the SARS-CoV-2 virus have put forth an immense pressure and urgent need for an effective and safe vaccine. The spike protein of SARS-CoV-2 is a potential target for vaccine development because of its role in virus-host receptor binding and membrane fusion, and because of its ability to elicit a neutralizing antibody response. The receptor binding domain of the spike protein is an independent entity as it folds independently, therefore, it is easy to produce. RBD has potential to generate both a humoral neutralizing antibody and a cellular immune response upon immunization. Therefore, RBD is an attractive target for SARS-CoV-2 vaccine development and has been used in many different vaccine development studies recently involving mRNA, DNA, or a protein subunit (47).

The sequence coding for the SARS-CoV-2 RBD was deduced through a structural-based sequence alignment based on the published structure of the SARS-CoV RBD; the identified sequences were further analyzed for potential glycosylation sites and folding specificity in terms of paired cysteine residues. The potential glycosylation site at N331 has been removed from many RBD constructs characterized previously in order to facilitate high protein yield (27, 48). However, deletion of the N331 and N343 glycosylation sites has been shown to drastically reduce infectivity, revealing an important role of glycosylation for viral infectivity (49). Glycosylation of the spike protein has been characterized previously for its role in the elicitation of potent neutralizing antibody response in comparison to the deglycosylated RBD (dsRBD), in terms of its importance as a vaccine candidate. RBD glycosylation sites are believed to mask non-neutralizing epitopes, whose exposure (non-neutralizing epitopes) in terms of glycan removal (engineered) or the expression of the deglycosylated form of protein (*E. coli*) is prone to elicit more non-neutralizing antibody responses (50). Pichia-based expression platforms lead to complex glycosylation, *bacullovirus* expression causes non-native glycan incorporation, and an *E. coli*-based expression system results in insoluble protein. The complex glycosylation of the insect cells differs from the native form produced by human cells and sometimes changes the immunogenic property of vaccine targets produced in those cell line (51), and re-solubilization or refolding in the *E. coli* system-based expressed protein always leaves the concern toward reaching the optimum functionality which requires the proper display of its neutralizing epitopes. Hence, mammalian cell lines are naturally fit for the production and secretion of the protein with precise glycosylation (52) and with the desired conformation. Glycosylation sites of RBD have been characterized to differentially mask (non-neutralizing)/unmask the epitopes, act as a conformation control element (50, 51) to maintain stability, solvent accessibility as well as its immunogenicity (53, 54). Therefore, we chose the non-truncated and wild-type sequences of the SARS-CoV-2 RBD as a vaccine candidate.

We expressed, purified, and characterized the RBD protein. The gel elution profile suggested monomeric conformation of the eluted protein, which is an advantage for a vaccine candidate as non-native oligomeric conformation sometimes masks the potential neutralizing epitope at the oligomerization interface leading to the antibody response targeting such an occluded epitope. Our deglycosylation studies show a reduction in protein size by approximately 6-7 kDa markedly highlighting the presence of two glycans. The peptide mass fingerprint with a Mascot score of 11499 and 35% sequence coverage of the expression sequences of RBD confirms the integrity of the expressed protein. The ability and binding specificity of RBD has been further characterized by its reactivity toward CR3022 and II62 (published elsewhere) (55) monoclonal antibodies and SARS/SARS-CoV-2 polyclonal sera. The ability of the RBD to interact with hACE2, the host cellular receptor was characterized by gel filtration chromatography, ELISA, and BLI-based studies, and were in agreement with previously reported parameters. As Streptavidin-Biotin is the strongest interaction, we biotinylated hACE2-His protein and deduced the RBD interaction in terms of dissociation constant, further hACE2 with Fc tagged capture *via* an AHC sensor also showed the presence of nanomolar affinities. The thermal stability of a vaccine candidate is an important parameter to facilitate its storage, shipment, and administration, RBD_330-526_ showed no loss in ACE2 binding specificity even at 40% loss in protein post 36 h at 37°C showing that it is an ideal vaccine candidate.

Efforts to develop safe and effective vaccines increasingly involve the use of adjuvants—substances formulated as part of a vaccine to boost immune responses and enhance the vaccine’s effectiveness with neutralizing antibodies, providing a degree of protection against viral challenges. For the immunization studies, C57BL/6 mice were selected for unbiased T helper response evaluation to further validate and correlate the outcome of the study in terms of hACE2 transgenic mice (56) in the future. RBD immunization without any adjuvant formulation was employed to study and compare the immunomodulatory effect of adjuvant candidates. RBD immunization alone showed a significant humoral and cellular response, but it was comparatively weaker relative to the adjuvant formulation. This however needs further validation in a challenge model of COVID-19, which should reveal the strength and duration of protection elicited by the RBD antigen in the absence of an adjuvant. Recently published literature of a COVID-19 vaccine candidate (ChAdOx1 nCoV-19) has shown that the immune response post vaccination was dominated by IgG1 and IgG3 subclasses (57). Similar correlations were observed in mice studies where the immune response was dominated by IgG1 and IgG3 antibodies in the RBD-adjuvant formulation groups followed by the IgG2 subclass. COVID-19 convalescent human sera have also shown that a very low spike or RBD-directed IgG2 and IgG4 isotype response is present post infection (58). The squalene-based AddaVax and alum-magnesium-based Imject RBD formulation elicit a high antibody titer and prominent neutralizing antibody response, which also reduces the probability of antibody-dependent enhancement (ADE), shown to be associated with suboptimal antibody response in COVID-19 (46) patients. Therefore, high affinity neutralizing antibody response as shown by BLI based on ACE2 competition and surrogate neutralization assays, with the Imject + RBD immunized group could significantly overshoot the threshold and mediate better protection. IL-17A or Th17 response is still uncertain and under investigation to ascertain whether it is a friend or foe post SARS-CoV-2 infection (59). The AddaVax + RBD and RBD alone formulation animal groups showed a slightly elevated IL-17A response in comparison to the Imject immunized group (**Figure 6B**). T cell responses analysis (antigen specific and nonspecific) showed good CD4+ and CD8+ T cell responses post immunization. The T cell mediated immune response of the RBD antigen showed a significantly upregulated frequency of IFN-γ secreting cells among the Imject + RBD group (**Figure 6**), a marker for Th1 response which promotes virus clearance. Similar results have also been reported by the COVID-19 RBD protein or mRNA-based vaccine candidates (60, 61).

The virus vector and subunit-based COVID-19 vaccine studies show a correlation between the reduction in immune enhancement through alum adjuvants (43). Though, alum as a vaccine adjuvant successfully induces antibody-mediated protective immunity [CoV-RBD219N1 (62) and PiCoVacc (9)], however, its ability to induce cellular immune response is limited. To overcome this limitation, in the ongoing vaccine development studies, alum is being used in combination with other additives/adjuvants to enhance cellular immune responses (63). The Imject formulation contains nonclinical magnesium as a component in addition to alum. Magnesium by itself modulates the immune system and inflammatory response; magnesium ions potentially inhibit macrophage activation by blocking certain calcium channels and contribute toward the anti-inflammatory effect that may divert the priming of pathogenic T cells toward a protective T cell response, which potentiates its further evaluation with clinically approved alum formulations. As adjuvants could direct the magnitude of the humoral and T cell response (64, 65), we observed that the RBD+AddaVax formulation promotes mixed IFN-γ (Th1) and IL-17 (Th17) responses, compared to the RBD+Imject response. Hence, based on our studies and literature supporting alum-based vaccine adjuvant formulations, we believe Imject is a better suited formulation for RBD-based vaccine candidates. Collectively, with these promising results including a high neutralizing antibody titer, antigen-specific CD4+ T cell response, significant cytotoxic CD8+ T cells, Th1 polarized IFN-γ elevation, and reduced Th-17 secretion, potentiate the efficacy of RBD (aa330-aa526) as a potential vaccine candidate. This study supports the further clinical and protective response evaluation of RBD_330-526_ as a vaccine candidate to satisfy the global need for a vaccine.

## Data Availability Statement

The original contributions presented in the study are included in the article/**Supplementary Material**. Further inquiries can be directed to the corresponding author.

## Ethics Statement

The animal study was reviewed and approved by Institutional Animal Ethics Committee (IAEC); IAEC/THSTI/93.

## Author Contributions

TS conceived and conceptualized the study. TS designed and performed experiments, analyzed data, and wrote the original draft. TS and BS purified the protein and carried out biochemical and biophysical experiments. SG assisted the protein purification work. RV performed the immunofluorescence microscopy and FACS studies. AA and ZR executed the cytokine expression experiment and analyzed the data. PV carried out animal immunization. SM performed SNT assays and KJ assisted the assay. SB executed the PRNT assays. SS assisted in the assay setup. MS contributed in data analysis and in the editing of the manuscript. All authors contributed to the article and approved the submitted version.

## Conflict of Interest

TS and SG are listed as inventors on the patent application; IPA No. - 202011018845 (LIPC Ref- IP0459).

The remaining authors declare that the research was conducted in the absence of any commercial or financial relationships that could be construed as a potential conflict of interest.
