# Supplementary material for: Comparative Immunomodulatory Evaluation of the Receptor Binding Domain of the SARS-CoV-2 Spike Protein; a Potential Vaccine Candidate Which Imparts Potent Humoral and Th1 Type Immune Response in a Mouse Model
Source: Front Immunol. 2021 May 24;12:641447. doi: 10.3389/fimmu.2021.641447 (PMC8182375; doi:10.3389/fimmu.2021.641447)
Supplement: Supplementary file 1 [file DataSheet_1.docx]

**Supporting Information (SI)**

**Comparative Immunomodulatory Evaluation of the Receptor Binding Domain of the SARS-CoV-2 Spike Protein; a Potential Vaccine Candidate Which Imparts Potent Humoral and Th1 Type Immune Response in a Mouse Model**

Tripti Shrivastava*^1^, Balwant Singh^1#^, Zaigham Abbas Rizvi^1#^, Rohit Verma^1^, Sandeep Goswami^1^, Preeti Vishwakarma^1^, Kamini Jakhar^1^, Sudipta Sonar^1^, Shailendra Mani^1^, Sankar Bhattacharyya^1^, Amit Awasthi^1^ and Milan Surjit^1^

^1^Infection and Immunology, Translational Health Science & Technology Institute, NCR Biotech Science Cluster, Faridabad, Haryana-121001, India

# BS and ZAR contributed equally

* Corresponding Author

Corresponding author mailing address:

Tripti Shrivastava, Ph.D,

Senior Research Scientist

Translational Health Science and Technology Institute (THSTI)

Correspondence: [tripti@thsti.res.in](mailto:tripti@thsti.res.in)

**Supplementary Information**

**Purification, Characterization and reactivity of RBD immunized sera with prefusion spike trimer (S2P)**

SARS CoV-2 S2P; prefusion stabilized ectodomain construct was gift from Barney S. Graham’s lab (NIH). The construct with 2P mutation, C-terminal T4 fibritin trimerization motif to stabilize prefusion trimeric conformation and Twin StrepTag and 8XHisTag (1), was expressed in fully glycosylated form through mammalian expression system (Expi 293 cells, Thermo Fisher) following the protocol used for expression of RBD. Briefly, Expi293 cells were transfected with plasmid DNA using ExpiFectamine 293 Transfection Kit (ThermoFisher). Transfected supernatant with secreted protein was harvested 5 days post-transfection by centrifugation of the transfected culture. The harvested supernatant with spike trimer loaded onto Ni-NTA agarose (Qiagen), equilibrated with 50mM Tris pH 7.4 and 100mM NaCl at 4 degrees temperature utilizing gravitational flow, the column was washed further with equilibration buffer and protein eluted with elution buffer containing 500mM Imidazole. NI-NTA purified fractions then pooled and dialyzed against PBS. The dialyzed spike trimeric protein further subdivided in small aliquots, snap-frozen in liquid nitrogen and stored at −80°C until further use.

The eluted factions from Ni-NTA column were checked on SDS PAGE and through western using anti SARS polyclonal antibody (Fig. S5A). Purified IgG from the pool sera from each immunized group was captured BLI sensors (to protein G) and its interaction with spike prefusion trimer was measured by immersing the sensor into spike solution followed by dissociation with the reaction buffer (Fig. S5B). Interaction and binding specificity of Spike trimer (S2P) which was used to measure the reactivity of RBD immunized sera on ELISA platform was also was validated with CR3022 and ACE2-Fc interaction (Fig. S5C).

**Expression and purification of ACE2-His and ACE2-Fc, biotinylation of ACE2-His**

The codon optimized expression constructs for ACE2-his and ACE2-Fc were expressed and purified from Expi293 cells transfected supernatant, following the similar protocol mention above. However the expressed supernatant of ACE2-Fc (Human IgG1-Fc) was incubated with Protein A resin for 1hr at 4ºC and passed through the empty Econo-Pac® chromatography columns. The resin washed with 1X PBS followed by wash with phosphate wash buffer containing 500mM NaCl. The protein eluted with 0.1M Glycine pH 2.8, the eluted protein immediately neutralize with 100mM Tris pH 8.0. The purity of the protein confirmed through SDS PAGE and pooled eluted fractions dialyzed against PBS (Fig. S3A). The dialyzed protein finally purified through Superdex 200 Increase 10/300 GL column (GE Healthcare), equilibrated in PBS to remove trace contaminants present if any. The purified ACE2-His and ACE2-Fc proteins were snap-frozen in liquid nitrogen and stored at −80°C in small aliquots until further use. The specificity of the purified ACE2 protein variants was confirmed with ACE2 Monoclonal Antibody (MA531395, Thermo Fisher Scientific) through western blot.

For ACE2-RBD affinity measurement, hACE2 his was biotinylated with EZ-Link NHS-PEG4-Biotin from Thermofisher following the manufacturer protocol. The biotinylated ACE2 from the biotinylation reaction was purified through gel filtration chromatography and stored at 4ºC in amber tubes till affinity measurement experiment.

**Peptide Mass fingerprinting**

The peptide mass fingerprinting was performed as per the methodology published by *Ingole et al.* (2) and *Lunge et al.* (3). Briefly; the protein gel was stained with Coomassie brilliant blue solution to confirm the desired band of interest, protein bands was excised from the gel using surgical blade, de-stained, washed, dehydrated and dried using speed-vac centrifuge at room temperature. Disulfide bonds were reduced and samples were incubated with Trypsin (Promega, USA) at final protease:protein ratio of 1:20 (w/w) for in-gel digestion at 37 °C for 16 h. Digested peptides were extracted using gradient of 20 % to 80 % ACN diluted in 0.1 % formic acid. Vacuum dried peptides were resuspended in 10 μl 2 % ACN in 0.1 % FA and subjected to MS/MS using TripleTOF^®^ 5600^+^ (ABsciex) mass spectrometer instrument attached with analytical column (ChromeXP, 3C18-CL-120). The peptides were eluted from the column and the data acquired through TF for MS and MS/MS analysis. The parent spectra were acquired with the scan range of 350-1250 m/z. intense peaks were fragmented using collision-induced dissociation with iTRAQ specific rolling collision energy in each cycle. The MS/MS spectra were acquired from 100 to 1600 Da.

Identification of peptides was performed by ProteinPilot software, version 5.0.1 (AB SCIEX) using the Paragon algorithm as the search engine. MS/MS spectra were searched against the SwissProt 2020_03 (562755 sequences; 202599198 residues) database. The peptide fragments analyzed with reference to SARS CoV-2 protein sequence with MASCOT score of 11499 (Figure S2C).

**Expression and purification of RBD-Fc**

RBD-Fc expressed and purified as per the methodology used for expression and purification of ACE2-Fc. The reactivity and binding specificity of RBD immunized sera as well as SARS/SARS CoV polyclonal sera with RBD-Fc was deduce through western blot analysis and at BLI platform. Supplementary figure 7A shows the equivalent reactivity of RBD-Fc as compared to RBD. Purified IgG from antigen immunized groups were capture on Protein G sensors and its reactivity to RBD-Fc was measured (Fig. S7 A), similarly in a separate set of experiment RBD-Fc was captured to the AHC sensors and its binding to immunized pool sera was measured.

**Supplementary figure legend:**

**Figure S1: Sequence alignment of SARS CoV-2 spike with closest matched structure identified from PDB blast.** Sequence alignment of SARS CoV-2 spike protein (QHO62107.1), with sequences from structure of SARS CoV showing closest homology; (2GHV (73.60%) (4), 2DD8 (74.11%) (5) and 3BGF (73.71%) (6). The sequence alignment was done through Clustal Omega and represented through Esprit script. The secondary structure assignment was done in reference to 7BWJ (7). The boundaries of the RBD expression construct designated by the dotted lines (330-526)

**Figure S2: Characterization of SARS CoV-2 RBD. (A)** SDS-PAGE showing RBD deglycosylation with increasing concentration of PNGase. **(B)** Detection of RBD with Anti SARS-pAb. **(C)** Peptide mass finger printing performed to validate the identity of purified protein bold underline sequences represent the identified peptide following trypsin digestion. **(D)** Dose dependent ELISA showing binding response of mAb CR3022 and **(E)** Anti SARS-pAb.

**Figure S3: Purification of hACE2-His and hACE2-Fc as well as binding response of hACE2-Fc to RBD: (A)** SDS PAGE showing purified band of hACE2-His and hACE2-Fc, western blot with anti ACE2 antibody confirms the protein specificity. **(B)** BLI based measurement of binding affinity of hACE2 with RBD used as analyte in various concentrations (sown in different colours), the Red solid line shows the fitted curve

**Figure S4: Characterization of RBD samples incubated over time at different temperature**. **(A)** Western blot of temperature dependent protein stability of RBD analyzed using anti-SARS polyclonal antibody. ELISA graph showing the dose dependent reactivity of end point incubated (72hrs for 4ºC, RT and 36hrs incubated sample at 37 ºC respectively) RBD with mAB CR3022 **(C)** and pAb **(D)**, the control graph represent relative activity of RBD at 0hr time point.

**Figure S5: Purification of Spike trimer and Functional characterization of RBD immunized sera (A)** SDS PAGE and western blot (anti-SARS polyclonal antibody) of purified spike trimer (S2P) recognizing band ~180kDa, **(B)** BLI based binding profile of purified IgG from each group pooled sera towards spike trimer **(C)** Dose dependent binding response of monoclonal antibody (i) CR3022 and (ii) hACE2-Fc respectively. **(D)** Binding antibody response of individual animal from the immunized groups toward RBD and Spike**:** Binding antibody titers of immunized sera from individual mice from each group post boost 2 towards RBD (left lane) and spike trimer (S2P) (right lane)

**Figure S6: Characterization of T-cell immune Responses (A)** *In vitro* PMA/Ionomycin-stimulation of Splenocytes, used for intracellular cytokine staining of IFNγ, IL-2 and IL-17A cytokines after CD4 and CD8 surface staining. Dot plots showing the population of respective T cell populations (Right panel) and Representative Bar graph (right panel) plotted for percent of positive cells ± standard errors of the mean (SEMs) for each group. **(B)** Sandwich ELISA of IFNγ, IL-17A and IL-10 cytokine of the *in vitro* PAM/Ionomycin stimulated culture soup was quantitated by using anti-mouse IFNγ, IL-17A or IL-10 primary and secondary antibodies.

**Figure S7: RBD Fc purification, reactivity towards RBD sera, PRNT and SNT to estimate neutralizing antibody titer. (A)** The RBD-Fc purified and its reactivity towards anti RBD sera from different group was measured through western blotting **(B)** PRNT assay with Anti-RBD sera from different immunized group, each sera dilution was performed in duplicate in 6 well plates. Maximum dilution tested for mouse sera from RBD + AddaVax group and RBD + Imject group does not reached to PRNT 90 or PRNT50 estimation. The control group (PBD, AddaVax and Imject) sera shows no reduction in the formation of plaques **(C)** Serum neutralization test with Anti-RBD sera from different immunized group was tested in duplication in 96 well format, well image from three consecutive well show the CP formation at (red circle) 1280, therefore neutralization titer 1:640 dilution. RBD+ AddaVax and ABD + Imject group shows neutralizing antibody titer higher than 1:5120 dilution (Red circle shows no CPE formation even at highest dilution used).

**Reference**

1. Wrapp D, Wang N, Corbett KS, Goldsmith JA, Hsieh CL, Abiona O, et al. Cryo-EM structure of the 2019-nCoV spike in the prefusion conformation. *Science (80- )* (2020) **367**:1260–1263. doi:10.1126/science.aax0902

2. Ingole KD, Dahale SK, Bhattacharjee S. Proteomic analysis of SUMO1-SUMOylome changes during defense elicitation in Arabidopsis. *bioRxiv* (2020)

3. Lunge A, Gupta R, Choudhary E, Agarwal N. The unfoldase ClpC1 of Mycobacterium tuberculosis regulates the expression of a distinct subset of proteins having intrinsically disordered termini. *J Biol Chem* (2020) **295**:9455–9473. doi:10.1074/jbc.RA120.013456

4. Hwang WC, Lin Y, Santelli E, Sui J, Jaroszewski L, Stec B, et al. Structural basis of neutralization by a human anti-severe acute respiratory syndrome spike protein antibody, 80R. *J Biol Chem* (2006) **281**:34610–34616. doi:10.1074/jbc.M603275200

5. Prabakaran P, Gan J, Feng Y, Zhu Z, Choudhry V, Xiao X, et al. Structure of severe acute respiratory syndrome coronavirus receptor-binding domain complexed with neutralizing antibody. *J Biol Chem* (2006) **281**:15829–15836. doi:10.1074/jbc.M600697200

6. Pak JE, Sharon C, Satkunarajah M, Auperin TC, Cameron CM, Kelvin DJ, et al. Structural Insights into Immune Recognition of the Severe Acute Respiratory Syndrome Coronavirus S Protein Receptor Binding Domain. *J Mol Biol* (2009) **388**:815–823. doi:10.1016/j.jmb.2009.03.042

7. Ju B, Zhang Q, Ge J, Wang R, Sun J, Ge X, et al. Human neutralizing antibodies elicited by SARS-CoV-2 infection. *Nature* (2020) **584**:115–119. doi:10.1038/s41586-020-2380-z
